# Genetic polymorphism of the *Nrf2* promoter region is associated with vitiligo risk in Han Chinese populations

**DOI:** 10.1111/jcmm.12874

**Published:** 2016-05-25

**Authors:** Pu Song, Kai Li, Ling Liu, Xiaowen Wang, Zhe Jian, Weigang Zhang, Gang Wang, Chunying Li, Tianwen Gao

**Affiliations:** ^1^ Department of DermatologyXijing HospitalFourth Military Medical UniversityXi'anChina

**Keywords:** vitiligo, SNP, *Nrf2*

## Abstract

The nuclear factor erythroid‐derived two‐like 2‐antioxidant response element (Nrf2‐ARE) pathway and its downstream antioxidant enzyme heme oxygenase‐1 (HMOX1 or HO‐1) play essential roles in H_2_O_2_‐induced oxidative damage in human melanocytes. However, the link between *Nrf2* promoter polymorphisms and susceptibility to oxidative stress‐related diseases such as vitiligo is unknown. This study evaluated the association of the *Nrf2* and *HO‐1* genes polymorphisms with vitiligo susceptibility. In this case–control study of 1136 Han Chinese vitiligo patients and 1200 controls, *Nrf2* (rs35652124 and rs6721961) and *HO‐1* (rs2071746) genes were genotyped by PCR‐restriction fragment length polymorphism analysis. Overall, a significantly decreased risk of vitiligo was found to be associated with *Nrf2* rs35652124 CC and combined (CT+CC) genotypes [odds ratio (OR) 0.64, 95% confidence interval (CI) 0.50–0.83 and OR, 0.84, 95% CI 0.71–0.99, respectively], as well as among subgroups: female, onset age ≤20 and never smoker. We subsequently found that *Nrf2* rs35652124 C allele had higher transcriptional activity in the luciferase reporter assay compared with *Nrf2* rs35652124 T allele. Furthermore, we investigated serum HO‐1 activity was associated with the rs35652124 CT+CC genotype and lower in patients than in controls (*P* = 0.024). Logistic regression analysis showed a dose–response relationship between lower vitiligo risk and increased HO‐1 activity in rs35652124 CT+CC genotype carriers (*P*_trend_ < 0.05). These findings indicate that the C allele of rs35652124 located in the promoter region of *Nrf2* gene is associated with protective effect on vitiligo in a Han Chinese population.

## Introduction

Vitiligo, an acquired skin depigmentation/hypopigmentation disorder, affects 0.05–1% of the global population and approximately 0.09% of the Chinese population, independently of gender 1, 2. The age of onset is typically before 20 years. Although the exact cause is unknown, vitiligo is thought to result from complex pathogenetic mechanisms involving a combination of environmental and genetic risk factors. Some studies have suggested that oxidative stress triggers the degeneration of basal layers of epidermal melanocytes 3, 4. Reactive oxygen species (ROS) disrupt the homoeostasis of melanocytes and damage nucleic acids, lipids and proteins, leading to cell death 5; for instance, excessive accumulation of H_2_O_2_ is observed in the epidermis of vitiligo patients and plays an important role in the disease 6, 7. Meanwhile, accumulating lines of evidence have also revealed that the nuclear factor erythroid‐derived 2‐like 2 (Nrf2) is a pivotal transcription factor of the antioxidant response in oxidative stress‐related illnesses 8.

Nrf2 protein, a member of the Cap‐N‐Collar basic leucine zipper transcription factor family, dimerizes with musculoaponeurotic fibrosarcomaor members of the activator protein 1 family in the cytoplasm. In response to oxidative stress, Nrf2 is quickly translocates into the nucleus and binds to the antioxidant response element (ARE), which is located in the upstream promoter region of many antioxidative genes 9. Therefore, the activation of Nrf2 is an important clue for the inducible expression of cytoprotective genes. Some phase‐detoxifying enzymes encoded by these genes, such as heme oxygenase‐1 (HMOX1 or HO‐1), glutathione S‐transferase (GST), NADH quinone oxidoreductase‐1 and superoxide dismutase be confirmed to play a major role in oxidative stress 10, 11. As one of the crucial cytoprotective proteins which induced by activation of Nrf2, HO‐1 has been shown to protect from some oxidative related pathologies, including hypertension, atherosclerosis and kidney injury 12, 13, 14. In addition, our previous study found that the Nrf2‐ARE pathway and its downstream antioxidant enzyme HO‐1 can mitigate H_2_O_2_‐induced oxidative damage in human melanocytes 15. Taken together, these findings indicated that involvement of altered Nrf2 expression may closely correlated with the pathogenesis of vitiligo.

**Figure 1:**
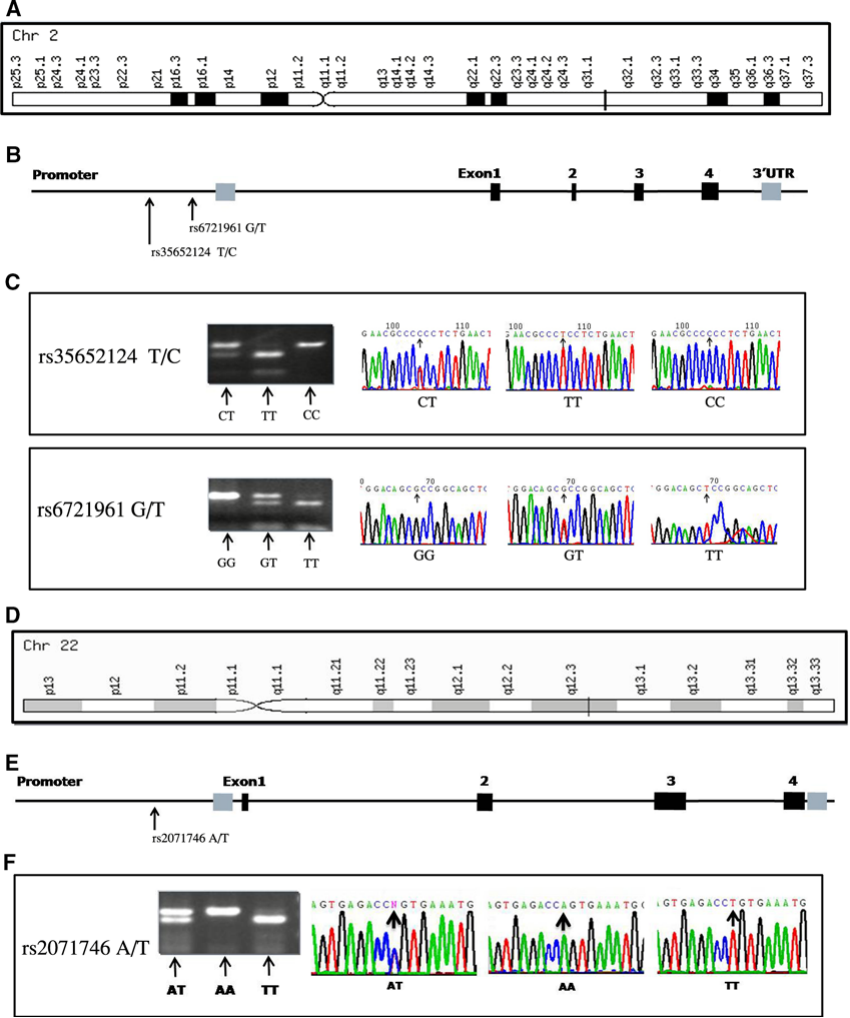
Location and detection of the *Nrf2* gene and *HO‐1* gene and SNPs. *Nrf2*, nuclear factor erythroid 2‐related factor 2; *HO‐1*, heme oxygenase‐1; SNPs, single nucleotide polymorphisms. (**A**) Location of the *Nrf2* gene. (**B**) The *Nrf2* gene structure and the location of the selected SNPs. (**C**) Genotypes of rs35652124 and rs6721961 in the *Nrf2* gene and sequence analyses of the *Nrf2* PCR products. (**D**) Location of the *HO‐1* gene. (**E**) The *HO‐1* gene structure and the location of the selected SNPs. (**F**) Genotypes of rs2071746 in the *HO‐1* gene and sequence analyses of the *HO‐1* PCR products.

The human *Nrf2* gene maps to chromosome 2q31 and consists of five exons and four introns (Fig. 1A). It was known that polymorphisms of the *Nrf2* gene had effects on the affinity and expression of the Nrf2 proteins and activation of the Nrf2 16, 17. Among the previous studies on single nucleotide polymorphisms (SNPs) of the *Nrf2* gene, the polymorphisms of *Nrf2* promoter region paly a major role in affecting its transcriptional activity. Two polymorphisms in the promoter region – a T to C substitution at position −653 (*Nrf2* rs35652124) and a G to T substitution at −617 (*Nrf2* rs6721961) – are located in an ARE‐like element and the myeloid zinc finger gene 1 binding site respectively (Fig. 1B) 18. Human *HO‐1* gene on chromosome 22q12 comprises five exons and four introns (Fig. 1D) 19. An A to T substitution at position −413 (*HO‐1* rs2071746) of the promoter is associated with Parkinson's and Alzheimer's diseases and aspirin resistance (Fig. 1E) 20, 21, 22. Given that the activation of *HO‐1* transcription by Nrf2 has a protective effect against a variety of pathologies, it is possible that *Nrf2* and *HO‐1* gene polymorphisms are associated with the development of vitiligo, although there have been no studies to date investigating this link. This study has been undertaken to fill this gap.

As polymorphisms of the *Nrf2* gene may influence Nrf2 function and expression of phase II metabolism genes, thereby affecting antioxidative targeting of melanocytes and increasing the risk of vitiligo. To test this hypothesis, we selected three SNPs from the Hapmap database with minor allele frequency >5% in Chinese population to evaluate the associations between the genetic variants in the *Nrf2* and *HO‐1* genes and vitiligo risk in Han Chinese subjects. Among these SNPs, we identified a protect‐associated *Nrf2* rs35652124 T/C polymorphism in the promoter region by using PCR‐restriction fragment length polymorphism (PCR‐RFLP) method. And we further assessed the *Nrf2* promoter activity related to −653T→C polymorphism by using transient transfections and luciferase assays. In addition, serum levels of HO‐1 and GST were measured in these subjects to evaluate potential associations between *Nrf2* variants and clinical manifestations of vitiligo.

## Materials and methods

### Study subjects

The study protocol was approved by the Clinical Ethics Committee of Xijing Hospital and the experiments were conducted according to the principles outlined by the Declaration of Helsinki. From August 2007 to January 2011, vitiligo patients (*n* = 1136) and vitiligo‐free control subjects (*n* = 1200) were recruited from Xijing Hospital, the Fourth Military Medical University. Only Han Chinese subjects (more than 90% of the Chinese population) were included to avoid genotype frequency variations among ethnic groups. Patients that were diagnosed by dermatologists and had undergone treatment in the preceding 6 months were excluded. Active vitiligo was defined as the appearance of new lesions or the enlargement of existing lesions in the 3 months before presentation in our study project. The vitiligo‐free controls were excluded if they had received blood transfusions in the last 6 months, or if they had other autoimmune diseases or other depigmentation disorders, such as piebaldism and albinism. A questionnaire was provided to obtain demographic and other information, and patients and controls were matched for age (±5 years), sex and ethnicity (Table 1). The response rate among participants was nearly 90%. Prior to recruitment, each participant provided written, informed consent and donated 5 ml of blood collected in heparinized tubes for genomic DNA extraction.

**Table 1:** Clinical characteristics of the selected variables in cases of vitiligo and controls

	Case, *n* (%); *n* = 1136	Control, *n* (%); *n* = 1200	*P* a
Age (years, ≤20/>20)	519 (45.7)/617 (54.3)	545 (45.5)/655 '(54.6)	0.896
Gender (male/female)	608 (53.5)/528 (46.5)	644 (53.7)/556 (46.3)	0.944
Onset age (years, ≤20/>20)	681 (59.9)/455 (40.1)		
Type (segmental/nonsegmental)	101 (8.9)/1035 (91.1)		
Stage (stable/active)	228 (20.1)/908 (79.9)		
Family history (yes/no)	174 (15.3)/962 (84.7)		
Autoimmunity disease (yes/no)	31 (2.7)/1105 (97.3)		
Smoking status (never smokers/ex‐smokers/current smokers)	651 (57.3)/94 (8.3)/391 (34.4)	647 (53.9)/95 (7.9)/458 (38.2)	0.169
Drinking status (never/ever)	1110 (97.7)/26 (2.3)	1164 (97.0)/36 (3.0)	0.285

a *P* values based on two‐sided chi‐square test.

### Genotyping

Genomic DNA was extracted from peripheral blood samples, using a DNA isolation kit (Tiangen, Beijing, China). DNA purity and concentration were evaluated by spectrophotometric measurements of absorbance at 260 and 280 nm. The PCR‐RFLP method was used to amplify *Nrf2* (rs35652124, rs6721961) and *HO‐1* (rs2071746) with the following forward and reverse primers: *Nrf2* rs35652124 T/C, 5′‐CCT TGC CCT GCT TTT ATC TC‐3′ and 5′‐CTT CTC CGT TTG CCT TTG AC‐3′; *Nrf2* rs6721961 G/T, 5′‐GAA AGG CGT TGG TGT AGG AG‐3′ and 5′‐GAA TGG AGA CAC GTG GGA GT‐3′; *HO‐1* rs2071746 A/T, 5′‐GTT CCT GAT GTT GCC CAC CAA GC‐3′ and 5′‐CTG CAG GCT CTG GGT GTG ATT TTG‐3′, which generated products of 264, 278, and 151 bp, respectively, that were digested with *Bse*RI (MBI Fermentas, Leon‐Rot, Germany), *Ngo*MIV and *Hind*III (both from New England Biolabs, Ipswich, MA, USA) restriction enzymes respectively. The fragments that were generated by the digestion were 192 and 72 bp for *Nrf2* rs35652124 T/C (Fig. 1C), 215 and 63 bp for *Nrf2* rs6721961 (Fig. 1C), and 20 and 131 bp for *HO‐1* rs2071746 (Fig. 1F). Results were confirmed by repeating the genotyping for >10% of randomly selected samples, yielding a concordance of 100%.

### Cell culture

Human normal melanocyte PIG1 cells (generously provided by Dr Caroline Le Poole, Loyola University Chicago, Chicago, IL, USA) were cultured in Medium 254 (Cascade Biologics, Portland, OR, USA) supplemented with human melanocyte growth supplement (Cascade Biologics), 5% foetal bovine serum (Gibco‐Invitrogen, Carlsbad, CA, USA) under a humidified atmosphere containing 5% CO_2_ at 37°C. This cell line was immortalized by retroviral introduction of the human papillomavirus type 16 E6 and E7 genes. Human embryonic kidney 293T cells were cultured in DMEM (Gibco‐Invitrogen) with 10% foetal bovine serum (Gibco‐Invitrogen) under a humidified atmosphere containing 5% CO_2_ at 37°C. This cell line was transformed by adenovirus E1A gene.

### Construction of reporter plasmids

*Nrf2* promoter‐luciferase reporter plasmids containing either rs35652124 T or C sequences were prepared by amplifying the 731 bp promoter region (from −738 to −7) using the primers 5′‐CCGCTCGAG ACCACTCTCCGACCTAAAGG‐3′ (forward) and 5′‐CCGAAGCTTCGTCGGCGGCTCCTCCGGGCTC‐3′ (reverse) that included *Xho*I and *Hind*III (both from Cnservice Invitrogen, Shanghai, China) restriction sites. Amplified fragments were sequenced to confirm that there were no errors in matched nucleotides and that the plasmid contained the correct allele. The fragments were cloned into the pGL3‐basic vector (Promega, Madison, WI, USA) after both were cut with *Xho*I and *Hind*III enzymes. Vectors were sequenced to confirm the orientation and integrity of the inserts.

### Transient transfections and luciferase assays

For the luciferase assay, 293T and PIG1 cells were seeded in 24‐well plates (1 × 10^5^ cells per well) and transfect with 0.8 μg of the recombinant pGL3 reporter vector with either −653 T or −653 C allele using Lipofectamine 2000 Transfection Reagent (Invitrogen, Carlsbad, CA, USA). As an internal control, all plasmids were co‐transfect with 0.02 μg pRL‐SV40 (Promega). The pGL3‐Basic vector without an insert and pGL3‐Control vector (Promega) were used as a negative control and a positive control respectively. Cells were collected 48 h after transfection, and cell lysates were prepared according to the manufacturer's instructions. Luciferase activity was measured with a Dual‐Luciferase Reporter Assay System (Promega) and normalized to the activity of pRL‐SV40 with the Renilla luciferase gene. Independent triplicate experiments were performed for each construct.

### Serum HO‐1 and GST enzymatic activity

Computer‐generated random numbers were used to select samples from patients with non‐segmental vitiligo and normal controls (*n* = 180 each) for serum HO‐1 and GST activity analysis. No statistically significant difference was observed between the 180 vitiligo patients and the total patient population in terms of epidemiologic features and genotype frequencies. Serum HO‐1 and GST activities were measured using kits (Xi Tang Biotechnology Co. Ltd, Shanghai, China and Cusabio Biotech Co. Ltd, Wuhan, China, respectively), according to the manufacturer's instructions. The absorbance of samples was measured at 450 nm.

### Statistical analysis

Differences in the frequency distributions of demographic variables, each allele and genotypes of *Nrf2* and *HO‐1* polymorphisms, and serum HO‐1 and GST activities between patients and controls were evaluated with the chi‐square test. The statistical analysis of the relationship between *Nrf2* rs35652124 T/C genotype and serum HO‐1 and GST activity in vitiligo patients was performed with GraphPad Prism 5 software (GraphPad, San Diego, CA, USA) with the unpaired *t*‐test. Hardy–Weinberg equilibrium of the genotype distribution of the controls was also tested by a goodness‐of‐fit chi‐square test. Associations between polymorphisms and vitiligo risk were determined by computing odds ratios (ORs) and 95% confidence intervals (CIs) from both uni‐ and multivariate logistic regression analyses, adjusting for the following subgroups: age, gender, stage, type, onset age, family history, related autoimmune disease status, and smoking and drinking status. For the luciferase reporter assay, differences in expression levels of the luciferase gene between constructs were evaluated by the Student's *t‐*test. Two‐tailed tests of statistical analyses were performed with SAS software version 9.1 (SAS Institute, Inc., Cary, NC, USA). Statistical significance was defined as *P* < 0.05.

## Results

## Characteristics of study subjects

This analysis included 1136 Han Chinese patients with vitiligo and 1200 controls (Table 1). Age and sex were well matched between patients and controls (*P* = 0.896 and *P* = 0.944, respectively). Patients were considered to have early‐onset vitiligo (n = 681, 59.9%) if the age of onset was prior to 20 years old. Of the patients with vitiligo, 101 (8.9%) had segmental vitiligo and 1035 (91.1%) had non‐segmental vitiligo; 908 (79.9%) had active vitiligo and 228 (20.1%) had stable vitiligo in the cohort. Patients with vitiligo were considered to have a family history (*n* = 174, 15.3%), if they had one or more first‐ to third‐degree relatives afflicted with the condition. In addition, 31 (2.7%) patients with vitiligo had an accompanying autoimmune disease such as hyperthyroidism, alopecia areata, diabetes, halo naevi or connective tissue disease. There were 651 (57.3%) never smokers, 94 (8.3%) ex‐smokers (had quit smoking for >1 year), and 391 (34.4%) current smokers among patients; and 647 (53.9%) never smokers, 95 (7.9%) ex‐smokers and 458 (38.2%) current smokers among controls. The frequency distributions of smoking status were similar between patients and controls (*P* = 0.169). The drinking status (defined as individuals who had consumed alcohol at least once in their lifetime) among patients and controls was 26 (2.3%) and 36 (3.0%) respectively (*P* = 0.285).

## Association between *Nrf2* and *HO‐1* genotypes and vitiligo risk

*Nrf2* and *HO‐1* genotype distributions and their associations with vitiligo risk for patients and controls are presented in Table 2. Genotype frequencies among control subjects were in Hardy–Weinberg equilibrium (*P* = 0.649 for *Nrf2* rs35652124 T/C; *P* = 0.406 for *Nrf2* rs6721961 G/T and *P* = 0.363 for *HO‐1* rs2071746 A/T). TT, CT and CC genotype frequencies for *Nrf2* rs35652124 were 33.4%, 49.3% and 17.3%, respectively, in controls and 37.3%, 50.4%, and 12.3%, respectively, in patients. The *Nrf2* rs35652124 variant C allele frequency was significantly lower among the vitiligo cases than among the controls (*P* = 0.002). When the TT genotype was used as the reference, a statistically significant decreased risk of vitiligo was associated with the CC (adjusted OR = 0.64; 95% CI = 0.50–0.83) and combined (CT+CC) genotype (adjusted OR = 0.84; 95% CI = 0.71–0.99), whereas the CT genotype was not associated with a decreased vitiligo risk (adjusted OR = 0.91; 95% CI = 0.76–1.09).

**Table 2:** Genotypic frequency of the *Nrf2* and *HO‐1* polymorphisms between cases and controls and their associations with the risk of vitiligo

Genotypes	Cases (*n* = 1136)		Controls (*n* = 1200)^a^		*P* b	Adjusted OR (95% CI)^c^
	*n*	%	*n*	%		
*Nrf2* rs35652124					0.002	
TT	424	37.3	401	33.4		1.00 (reference)
CT	572	50.4	592	49.3		0.91 (0.76–1.09)
CC	140	12.3	207	17.3		0.64 (0.50–0.83)
CT+CC	712	62.7	799	66.6	0.048	0.84 (0.71–0.99)
C allele		35.7		40.7	0.002	
*Nrf2* rs6721961					0.447	
GG	508	44.7	561	46.8		1.00 (reference)
GT	508	44.7	528	44.0		1.06 (0.90–1.26)
TT	120	10.6	111	9.2		1.19 (0.90–1.59)
GT+TT	628	55.3	639	53.2	0.346	1.09 (0.92–1.28)
T allele		49.1		45.4	0.221	
*HO‐1* rs2071746					0.124	
AA	499	43.9	520	43.3		1.00 (reference)
AT	465	40.9	529	44.1		0.92 (0.77–1.09)
TT	172	15.0	151	12.6		1.19 (0.92–1.53)
AT+TT	637	56.1	680	56.7	0.773	0.98 (0.83–1.15)
T allele		55.3		53.0	0.482	

*Nrf2*, Nuclear factor erythroid 2‐related factor 2; *HO‐1*, Heme Oxygenase‐1; OR, odds ratio; CI, confidence interval.

a The observed genotype frequencies among the controls were in agreement with the Hardy–Weinberg equilibrium (χ^2^ = 0.207, *P* = 0.649 for *Nrf2* rs35652124; χ^2^ = 0.691, *P* = 0.406 for *Nrf2* rs6721961; χ^2^ = 0.828, *P* = 0.363 for *HO‐1* rs2071746).

b Two‐sided chi‐square test for distributions of genotype and allele frequencies between the cases and controls.

c Adjusted ORs were obtained from a multivariate logistic regression with adjustment for age and gender.

However, there was no evidence that the allele and genotype frequencies of *Nrf2* rs6721961 G/T and *HO‐1* rs2071746 A/T were associated with susceptibility to vitiligo (*P* = 0.447 for *Nrf2* rs6721961; *P* = 0.124 for *HO‐1* rs2071746).

### Effects of selected *Nrf2* variables in different subgroups and their association with vitiligo risk

To evaluate the effects of *Nrf2* polymorphism on patient characteristics, stratification analyses of *Nrf2* polymorphisms and vitiligo risk were performed (Table 3). The frequency of the *Nrf2* rs35652124 CT+CC genotype was lower for the subgroups, including female, onset age ≤20 years and never smokers in patients but not in controls (*P* = 0.029, *P* = 0.001 and *P* = 0.013 respectively). When *Nrf2* rs35652124 TT genotype was used as a reference, the CT+CC genotype frequency was lower among patient subgroups with the following characteristics: female (adjusted OR = 0.76, 95% CI = 0.59–0.97), onset age ≤20 years (adjusted OR = 0.65, 95% CI = 0.51–0.83) and never smokers (adjusted OR = 0.73, 95% CI = 0.57–0.94).

**Table 3:** Stratification analysis of the *Nrf2* rs35652124 genotypes and vitiligo risk by selected variables

Variables	*Nrf2* rs35652124 (case–control)				*P* a	Adjusted OR (95% CI)^b^
	TT		CT+CC			
	*n*	%	*n*	%		
Sex						
Male	199/200	17.5/16.7	409/444	36.0/37.0	0.525	0.93 (0.73–1.15)
Female	225/201	19.8/16.7	303/355	26.7/29.6	0.029	0.76 (0.59–0.97)
Onset age (years)						
≤20	228/184	20.1/15.3	291/361	25.6/30.1	0.001	0.65 (0.51–0.83)
>20	196/217	17.3/18.1	421/438	37.0/36.5	0.604	1.06 (0.84–1.35)
Type						
Segmental	38/401	3.3/33.4	63/799	5.6/66.6	0.390	0.83 (0.55–1.27)
Non‐segmental	386/401	34.0/33.4	649/799	57.1/66.6	0.056	0.84 (0.71–1.00)
Stage						
Stable	90/401	7.9/33.4	138/799	12.2/66.6	0.078	0.77 (0.58–1.03)
Active	334/401	29.4/33.4	574/799	50.5/66.6	0.108	0.86 (0.72–1.03)
Family history						
Yes	69/401	6.1/33.4	105/799	9.2/66.6	0.105	0.76 (0.55–1.06)
No	355/401	31.3/33.4	607/799	53.4/66.6	0.091	0.86 (0.70–1.01)
Autoimmunity disease						
Yes	15/401	1.3/33.4	16/799	1.4/66.6	0.082	0.54 (0.26–1.09)
No	409/401	36.0/33.4	696/799	61.3/66.6	0.071	0.85 (0.72–1.02)
Smoking status						
Never‐smokers	184/144	16.2/12.0	467/503	41.1/41.9	0.013	0.73 (0.57–0.94)
Ex‐smokers	60/57	5.3/4.7	34/38	3.0/3.2	0.588	0.85 (0.47–'1.53)
Current‐smokers	180/200	15.8/16.7	211/258	18.6/21.5	0.489	0.91 (0.69–1.19)
Drinking status						
Never	404/380	35.6/31.7	706/784	62.1/65.3	0.060	0.85 (0.71–1.01)
Ever	20/21	1.8/1.7	6/15	0.5/1.3	0.127	0.42 (0.14–1.29)

*Nrf2*, Nuclear factor erythroid 2‐related factor 2; OR, odds ratio; CI, confidence interval.

a Two‐sided chi‐square test for distributions of genotype and allele frequencies between the cases and controls.

b Adjusted ORs were obtained from a multivariate logistic regression with adjustment for age and gender.

### Effects of *Nrf2* rs35652124 polymorphism on transcriptional activity

**Figure 2:**
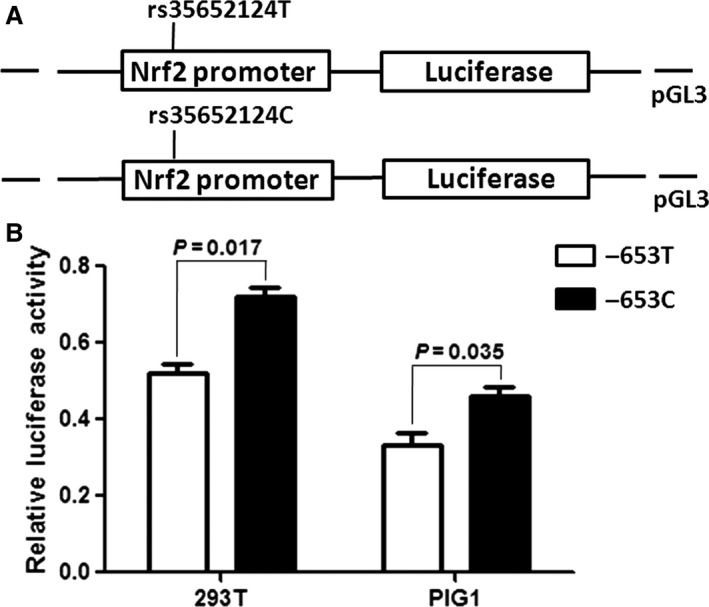
Effect of the rs35652124 T/C polymorphism in the *Nrf2* promoter activity. *Nrf2*, nuclear factor erythroid 2‐related factor 2. (**A**) Schematic representation of reporter plasmids containing the rs35652124T or rs35652124C allele, which was inserted upstream of the luciferase reporter gene in the pGL3 basic plasmid. (**B**) Two constructs were transiently transfected into the 293 T and PIG1 cells, respectively. The luciferase activity of each construct was normalized against the internal control of Renilla luciferase. Columns, mean from two‐independent experiments; bars, SD. *P < 0.05 compared with the construct counterpart.

To evaluate the promoter activity associated with *Nrf2* rs35652124 T/C polymorphism, T or C promoter constructs were transiently transfect into human embryonic kidney 293T and human normal melanocyte PIG1 cells. A higher relative luciferase activity was observed for the C than for the T allele of rs35652124 in both cell lines (*P* = 0.017 for 293T cells, *P* = 0.035 for PIG1 cells) (Fig. 2). These results indicate that the T to C transition in the *Nrf2* rs35652124 promoter region increases the transcriptional activity of the *Nrf2* gene.

### Serum HO‐1 and GST activities in vitiligo patients and controls

**Figure 3:**
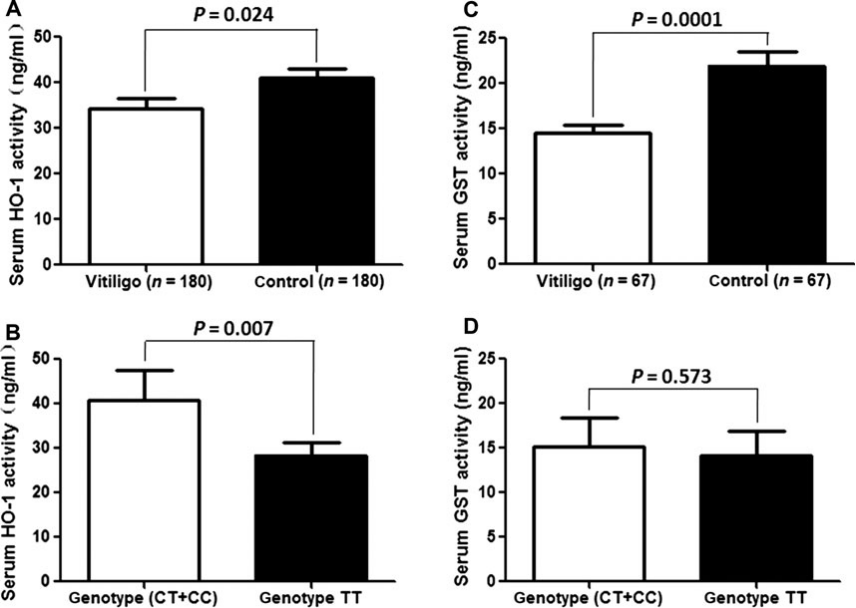
Serum HO‐1 activity and correlations to vitiligo genotype. *Nrf2*, nuclear factor erythroid 2‐related factor 2; *HO‐1*, Heme Oxygenase‐1. (**A**) The serum HO‐1 activity in vitiligo patients’ group is significantly lower than that in the normal control group (*P* < 0.05). (**B**) Compared with the *Nrf2* rs35652124 combined TT genotype group, the protect combined genotype CT+CC group has the high serum HO‐1 activity (*P* < 0.05). (**C**) The serum GST activity in vitiligo patients’ group is significantly lower than that in the normal control group (*P* < 0.05). (**D**) There were no differences in serum GST activity among *Nrf2* rs35652124 genotypes (*P* > 0.05).

Serum HO‐1 activity was measured in 180 vitiligo patients and 180 normal controls, whose demographic, clinical characteristics and genotype frequencies corresponded to those of the study population (1136 cases and 1200 controls; data not shown). As shown in Fig. 3A, HO‐1 activity was lower in the vitiligo than in the control group (34.91 ± 12.12 ng/ml *vs*. 40.97 ± 14.91 ng/ml; *P* = 0.024). Similarly, serum GST activity was lower in 67 vitiligo patient samples than in age‐ and sex‐matched control samples (14.50 ± 8.31 ng/ml *vs*. 21.94 ± 11.76 ng/ml; *P* = 0.0001) (Fig. 3C).

Furthermore, an analysis of the relationship between *Nrf2* rs35652124T/C genotype and serum HO‐1 activity in vitiligo patients showed that compared to TT, the combined genotype (CT+CC) genotype was associated with higher serum HO‐1 activity (*P* = 0.007) (Fig. 3B). However, there were no differences in serum GST activity among *Nrf2* rs35652124 genotypes (*P* = 0.573) (Fig. 3D).

### Logistic regression analysis of HO‐1 activity in vitiligo patients and controls

In a logistic regression analysis in which serum HO‐1 activity was dichotomized by median activity of the control group, a higher activity was associated with a 0.64‐fold reduction in vitiligo risk (*P* = 0.034, 95% CI = 0.42–0.97) (Table 4). Furthermore, when HO‐1 activity was divided into tertiles, according to the activity of the control group, a dose–response relationship between increased activity and decreased risk was apparent: suboptimal (upper tertile), intermediate (mid tertile) and efficient (lower tertile) activities had adjusted ORs of 1.00, 0.55 (*P* = 0.015, 95% CI = 0.33–0.90) and 0.43 (*P* = 0.001, 95% CI = 0.26–0.72) respectively.

**Table 4:** Logistic regression analysis of HO‐1 activity in vitiligo patients and controls

HO‐1 activity (ng/ml)	Case (*n*=180)		Control (*n*=180)		*P* a	Adjusted OR (95% CI)^b^
	*n*	%	*n*	%		
By median						
<32.1	110	61.1	90	50.0		1.00 (reference)
≥32.1	70	38.9	90	50.0	0.034	0.64 (0.42–0.97)
By tertile						
≤26.3	91	57.2	59	32.8		1.00 (reference)
26.3–43.6	50	10.6	60	33.3	0.015	0.55 (0.33–0.90)
≥43.6	39	32.2	61	33.9	0.001	0.43 (0.26–0.72)
Trend test						c *P* < 0.05

*Nrf2*, Nuclear factor erythroid 2‐related factor 2; *HO‐1*, Heme Oxygenase‐1; OR, odds ratio; CI, confidence interval.

a Two‐sided chi‐square test for distributions of genotype and allele frequencies between the cases and controls.

b Adjusted ORs were obtained from a logistic regression with adjustment for age and gender.

c Adjusted for age and sex.

### Association between *Nrf2* rs35652124 genotype and vitiligo risk based on HO‐1 activity

The association between vitiligo risk and *Nrf2* rs35652124 genotypes was examined with respect to HO‐1 activities (Table 5). *Nrf2* rs35652124 genotypes were divided into two categories: the 1‐2 (CT or TT) and 0 (CC) risk alleles. When carriers of the 1‐2 risk allele and with lower HO‐1 activity (≤32.1 pg/ml) were used as a reference, individuals with the 0 risk allele and higher HO‐1 activity (>32.1 pg/ml) had a decreased risk of vitiligo (adjusted OR = 0.43, 95% CI = 0.23–0.79). Consistent with these results, when the serum HO‐1 activity was divided into tertiles, individuals with the 0 risk allele and HO‐1 activity in the upper tertile (>43.6 pg/ml) had a larger decrease in vitiligo risk (adjusted OR = 0.41, 95% CI = 0.20–0.85) compared to those with the 1‐2 risk allele and lower tertile HO‐1 activity (≤26.3 pg/ml).

**Table 5:** Risk of vitiligo associated with *Nrf2* rs35652124 genotypes by HO‐1 activity

HO‐1 activity (pg/ml)	*Nrf2* rs35652124 (case–control)					
	CT/TT (1‐2 risk genotype)	*P* a	OR (95% CI)	CC (0 risk genotypes)	*P* a	OR (95% CI)
By median						
<32.1	64/47		1.00 (reference)	46/43	0.399	0.79 (0.45–1.38)
≥32.1	43/44	0.249	0.72 (0.41–1.26)	27/46	0.006	0.43 (0.23–0.79)
By tertile						
≤26.3	42/32		1.00 (reference)	49/29	0.446	1.29 (0.67–2.47)
26.3–43.6	30/33	0.286	0.69 (0.35–1.36)	20/27	0.128	0.56 (0.27–1.18)
≥43.6	20/26	0.157	0.59 (0.28–1.23)	19/35	0.016	0.41 (0.20–0.85)

*Nrf2*, Nuclear factor erythroid 2‐related factor 2; *HO‐1*, Heme Oxygenase‐1; OR, odds ratio; CI, confidence interval.

a Two‐sided chi‐square test for distributions of genotype and allele frequencies between the cases and controls.

ORs were obtained from a multivariate logistic regression with adjustment for age and gender.

## Discussion

Although the aetiology of vitiligo is not fully understood, the accumulation of H_2_O_2_ because of oxidative stress may be a trigger for melanocyte degeneration 23. Nrf2 is a redox‐sensitive transcription factor that has a protective role against oxidative stress, and *Nrf2* variants may have a influential capacity for binding promoters, which undermines target gene expression 24.

This study demonstrated that whether certain genetic polymorphisms would contribute to the risk of developing vitiligo. In 2008, Guan *et al*. found that an *Nrf2* promoter SNP at −650 position was associated with the development of vitiligo in China and the −650 A allele may be one of the risk factor 25. Their study provided genetic evidence for the relationship between *Nrf2*, an important antioxidant gene, and vitiligo. In addition to the −650 SNP loci, some other SNPs of human *Nrf2* have been shown to be associated with the risk of various diseases, which prompted us to evaluate their association with vitiligo susceptibility in this study. As a result, we demonstrated that a statistically decreased risk of vitiligo was associated with the *Nrf2* rs35652124 variant C allele, although no evident risk was associated with *Nrf2* rs6721961 or *HO‐1* rs2071746 variants, indicating that there was an intrinsic linkage between *Nrf2* genetic variants and the risk of vitiligo. Moreover, a lower vitiligo risk was found in the female, early‐onset age and never smoker subgroups. Since smoking known to aggravate oxidative stress, our result supported *Nrf2* gene polymorphism may play a key role for the systemic response to ROS in cigarette smoke exposure. Interestingly, the T to C substitution in *Nrf2* rs35652124 increased the transcriptional activity of Nrf2 *in vitro*, and a higher serum HO‐1 activity was detected in vitiligo patients with the CT+CC rather than the TT genotypes. These data suggested that this variant of *Nrf2* might affect Nrf2 function and the risk of developing vitiligo in a Han Chinese population.

The rs35652124 T/C polymorphism is located at position −653 of the *Nrf2* gene. The transcription factor binding search analysis was performed with TRANSFAC^®^ 7.0 public database (http://www.gene-regulation.com/cgi-bin/pub/programs/alibaba2/webbaba2.cgi) to ascertain the potential functional relevance of this SNP. Interestingly, this analysis was performed that the variant of *Nrf2* rs35652124 T/C is situated in the core sequence of the putative binding site for the transcription factor, specificity protein 1 (Sp1); this T to C allele mutation might therefore contribute to the combination of Sp1 with the *Nrf2* promoter region. Based on the *in vitro* reporter assay, the protective C allele of this polymorphism may enhance *Nrf2* promoter activity through influencing the binding activity of transcriptional regulator Sp1 compared with T allele. Thus, the *Nrf2* rs35652124 C allele may be expected to affect Nrf2 expression and the important regulatory roles in various cellular responses following oxidative damage, thereby conferring protection against vitiligo. This hypothesis is strongly supported by our results.

Oxidative stress activates transcription of a variety of genes encoding antioxidative enzymes through a cis‐acting sequence known as the ARE 26. The ARE is initially found in promoter regions of genes encoding phase II detoxification enzymes and antioxidant proteins 27. *Nrf2* rs6721961 G/T polymorphism is located at position −617 of the promoter region, affecting the ARE binding site and possibly gene expression. *Nrf2* functional polymorphisms have been linked to the risk of developing ALI after major trauma 28, childhood asthma caused by air pollution 29, and oxidative stress‐related defects in human spermatogenesis caused by heavy smoking 30. In addition, in one study of forearm vasodilatory responses, Caucasian subjects with the *Nrf2* rs6721961 T allele had decreased blood flow and increased vascular resistance as compared to that in non‐carriers, while no such association was observed among African‐American subjects 31, suggesting an ethnic component to the distribution of the *Nrf2* rs6721961 G/T polymorphism. In this study, there was no statistical correlation between rs6721961 and vitiligo risk. This may be due to sample size or variability across diseases and populations. Similarly, there was no evidence that *HO‐1* rs2071746 A/T is associated with susceptibility to vitiligo.

It has been suggested that oxidative stress may be an important contributor to the pathogenesis of melanocyte death because of the increase in the production of oxygen free radicals and a deficiency in antioxidant defences mechanisms. Many studies have examined the status of antioxidants and antioxidant enzymes in vitiligo patients 32. Meanwhile, up‐regulation of HO‐1 may represent an attempt to minimize cellular injury 33. In our previous study, we investigated the serum HO‐1 activity in patients with vitiligo and controls, and a lower HO‐1 activity was observed in vitiligo patients than in controls. This finding suggested that a deficiency in HO‐1 might contribute to the development of vitiligo. Moreover, HO‐1 is a major antioxidant enzyme regulated by Nrf2 in vitiligo, which plays a role in protecting melanocytes against H_2_O_2_‐induced oxidative stress. Induction of these antioxidant enzymes is mediated largely by the transcription factor Nrf2 34. Among patients with vitiligo, we report for the first time that there was a dose–response relationship between HO‐1 activity and *Nrf2* rs35652124 T/C genotype. Comprehensive analysis of HO‐1 activity and *Nrf2* rs35652124 T/C genotypes suggested that, much as with antioxidants, the rs35652124 C variant genotype played an important role in protection from oxidative damage. Individuals with higher HO‐1 activity and the rs35652124 C variant genotype might have a minor risk for developing vitiligo compared with those of low HO‐1 activity and an rs35652124 T wild genotype. Consistent with this hypothesis, targeted disruption of *Nrf2* changed antioxidant capacity in mice and thus increased susceptibility to pro‐oxidant and carcinogenic agents 35, 36.

Moreover, GSTs are a large family of enzymes participating in detoxification of endogenous. GST antioxidant activity in blood was proven to be linked to the risk of oxidative stress‐related type 2 diabetes mellitus and impaired glucose tolerance 37. These findings have led to the hypotheses that serum GST activity may be associated with vitiligo. We evaluated serum GST level, and a lower GST activity indeed existed in vitiligo patients than in controls. However, our results showed no association between serum GST and *Nrf2* rs35652124 polymorphisms. This is not surprising, since many cytoprotective genes which could regulate the enzyme production and, a single abnormality, may not be adequate to affect the level of GST activity. The population‐based studies with a larger sample size are necessary to confirm these findings.

In summary, we report that the *Nrf2* rs35652124 T/C polymorphism and serum HO‐1 activity affect susceptibility to vitiligo among Han Chinese population. Future studies will examine SNPs in other genes involved in oxidative stress to confirm the link between this process and melanocyte production in vitiligo. The present findings indicate that treatments strategies that regulate HO‐1 activity could potentially reverse melanocyte degeneration and depigmentation in vitiligo, ultimately leading to improved self‐esteem and better quality of life in patients. Furthermore, studies are necessary to evaluate whether other potential transcriptional mechanisms are associated with *Nrf2* rs35652124 T/C polymorphisms.

## Conflict of interest

The authors confirm that there are no conflicts of interest.
